# Overexpression of the Wild Soybean R2R3-MYB Transcription Factor *GsMYB15* Enhances Resistance to Salt Stress and *Helicoverpa Armigera* in Transgenic *Arabidopsis*

**DOI:** 10.3390/ijms19123958

**Published:** 2018-12-09

**Authors:** Xin-Jie Shen, Yan-Yan Wang, Yong-Xing Zhang, Wei Guo, Yong-Qing Jiao, Xin-An Zhou

**Affiliations:** 1 Key Laboratory of Biology and Genetic Improvement of Oil Crops, Ministry of Agriculture, Oil Crops Research Institute, Chinese Academy of Agricultural Sciences, Wuhan 430062, China; yabofei1212@163.com (Y.-Y.W.); yongxing1008@163.com (Y.-X.Z.); guowei@caas.cn (W.G.); 2 Graduate School of the Chinese Academy of Agricultural Science, Beijing 100081, China

**Keywords:** R2R3-MYB transcription factor, wild soybean, *Helicoverpa armigera*, salt stress, **Arabidopsis* thaliana*

## Abstract

Plant R2R3-MYB transcription factors (TFs) have been suggested to play crucial roles in the response to diverse abiotic and biotic stress factors but there is little molecular evidence of this role in soybean plants. In this work, we identified and functionally characterized an R2R3-MYB TF, namely, *GsMYB15*, from the wild soybean ED059. Protein and promoter sequence analysis indicated that *GsMYB15* is a typical R2R3-MYB TF and contains multiple stress-related *cis-*elements in the promoter region. *GsMYB15* is located in the nucleus and exhibits transcriptional activation activity. QPCR assays suggested that the expression of *GsMYB15* could be induced by NaCl, insect attacks and defense-related hormones (MeJA and SA). Furthermore, *GsMYB15* exhibited highest expression in pods compared to other tissues. Functional analysis of *GsMYB15* demonstrated that overexpression of *GsMYB15* could increase salt tolerance and enhance the resistance to *H. armigera larvae* in transgenic *Arabidopsis* plants. Moreover, overexpression of *GsMYB15* also affected the expression levels of salt stress- and defense-related genes in the transgenic plants. Feeding with transgenic *Arabidopsis* plant leaves could significantly suppress the expression levels of immunity-related genes in *H. armigera larvae*. Overexpression of *GsMYB15* also increased mesophyll cell levels in transgenic plants. Taken together, these results provide evidence that *GsMYB15* is a positive regulator of salt stress tolerance and insect resistance in transformed *Arabidopsis* plants.

## 1. Introduction

As sessile organisms that, unlike animals, cannot escape, plants have evolved sophisticated regulatory mechanisms for the maintenance of growth and development under diverse environmental stress conditions [1,2,3]. An increasing number of studies have suggested that plants can survive under different stress conditions, most likely by controlling complex plant hormone response networks and molecular signal transduction pathways [4,5,6]. When they encounter stressful conditions, plants first perceive stress signals via receptors and then exhibit specific changes in gene expression, protein synthesis, metabolic secretion and even physiological structure, leading to stress tolerance [7,8,9]. Among these molecular and physiological factors, the regulatory genes in the nucleus and the encoded proteins are critical for stress response and the functions of these biomolecules can be amplified via signal transduction cascades [5,10,11].

Plant growth and development are mainly regulated by six major hormones or groups of hormones, namely, abscisic acid (ABA), auxin (IAA), gibberellins (GAs), cytokinins (CTKs), ethylene (ET) and brassinolides (BRs). Although their levels are very low in plants, plant hormones play essential roles throughout the plant growth period. In addition to functioning as growth regulators, plant hormones also play important roles in the response to multiple environmental stress factors by acting as stress signaling molecules to transmit stress signals to downstream response genes [4,6,10]. The ABA signaling pathway is central to drought and salt stress responses in plants [6]. When plants encounter stressful conditions, the ABA receptors PYLs can sense signals and form the PYL-ABA-PP2C complex and the PP2C-SnRK complex dissociates, leading to the activation of downstream stress-related gene expression by SnRKs [12]. Recent research has also demonstrated that ABA can enhance plant pathogen immunity by regulating stomatal closure via the *AtPeps*-PEPR signaling pathway [13], indicating that ABA probably has multiple functions and can participate in crosstalk with other plant hormone pathways. Jasmonic acid (JA) and salicylic acid (SA) are the major defense hormones involved in the activation of defensive responses against herbivorous insects and pathogens, respectively [14,15,16]. When plants are injured by insect attacks, the wound signal stimulates plant JA synthesis and the JA signal then triggers the COI1-JAZ interaction, which leads to the degradation of JAZs by the 26S proteasome and to activation of downstream defense-related gene expression, such as the expression of MYC transcription factors (TFs) [17]. A recent study demonstrated that the ET-signaling pathway plays a central role in regulating the resistance to corn leaf aphids (CLAs) in *Zea mays* leaves but this mechanism is independent of JA [18]. BRs have also been reported to play essential roles in plant-herbivorous insect interactions due to the diverse secondary metabolites of BRs, such as cholesterol; insects must obtain sterol precursors from plants for de novo synthesis and these compounds play a crucial role in insect growth [19]. GAs and CTKs often act as nodes of crosstalk among different plant hormones in the stress response network [20]. For instance, the DELLA proteins can interact with JAZ1 to downregulate downstream defense-related gene expression of the JA signaling pathway [21]. The CTK-activated factor ARR2 can interact with TGA3 to promote gene expression of the SA signaling pathway gene expression, leading to activation of an NPR1-mediated defense mechanism [22].

A growing number of studies have shown that TFs play central roles in regulating gene expression in response to abiotic and biotic stress factors, such as low temperatures, salt, drought and pathogen and insect attacks [3,23,24,25,26,27]. The plant MYB TFs have been demonstrated to be involved in the response to various environmental stress factors and increase plant stress tolerance by activating downstream stress-related signal transduction pathways [3,28,29]. There are four types of MYB TFs in plants, namely, R1-, R2R3-, R1R2R3- and 4R-MYB, based on the number of DNA-binding domains [28]. The plant R2R3-MYB TF family has been widely studied in many plants and has been shown to be involved in stress response. In addition, increasing evidence suggests that R2R3-MYB TFs likely have multiple functions in the regulation of different stress responses. Heterologous expression of *PacMYBA* in *Arabidopsis* plants can enhance salt tolerance and pathogen resistance [3]. Overexpression of *TaMYB1D* in tobacco plants can increase the tolerance to drought and oxidative stress by affecting phenylpropanoid metabolism [30]. *AtMYB96* can regulate cuticular wax biosynthesis under drought conditions, leading to increased plant drought tolerance [23,31]. *AtMYB96* can also enhance pathogen resistance by promoting SA biosynthesis in **Arabidopsis*.* In addition to improving abiotic stress tolerance and pathogen resistance, plant R2R3-MYB TFs also play important roles in the defensive response to insects. *TaMYB19*, *TaMYB29* and *TaMYB44* are co-regulators of phloem-based defense against English grain aphids in wheat [32]. *AtMYB75* can modulate flavonoid metabolites, leading to resistance against *Pieris brassicae* via regulation of kaempferol-3,7-dirhamnoside biosynthesis [33].

Soybean is one of the most important oil crops in the world. Soybean not only produces cooking oil but also produces high-quality vegetable protein, which are benefic for human health [34]. As global warming increases and indiscriminate use of pesticides, the yield of soybean often face extremely environment conditions and fatal damage by insects [35]. Using molecular biology techniques could create GM soybeans which significantly increase the stress tolerance and yield of soybean [36]. However, the candidate genes using for improving soybeans are still limited. Plant R2R3-MYB TFs have been suggested playing crucial roles in regulating and improving plant in response to multiple abiotic and biotic stresses [2,3,37]. Identify and function analysis more R2R3-MYB TFs might benefit for soybean molecular breeding.

Although the functions of plant R2R3-MYB TFs in stress response have been widely studied in many plants, the role of R2R3-MYB TFs in salt tolerance and insect resistance in soybean plants remains unclear. In this work, we report a wild soybean R2R3-MYB TF, named *GsMYB15* and demonstrate that *GsMYB15* is a nuclear protein with transcriptional activation activity. Moreover, we showed that *GsMYB15* plays a positive role in the response to salt stress and insect resistance in transgenic *Arabidopsis* plants.

## 2. Results

### 2.1. Clone and Sequence Analysis of GsMYB15

**Figure 1:**
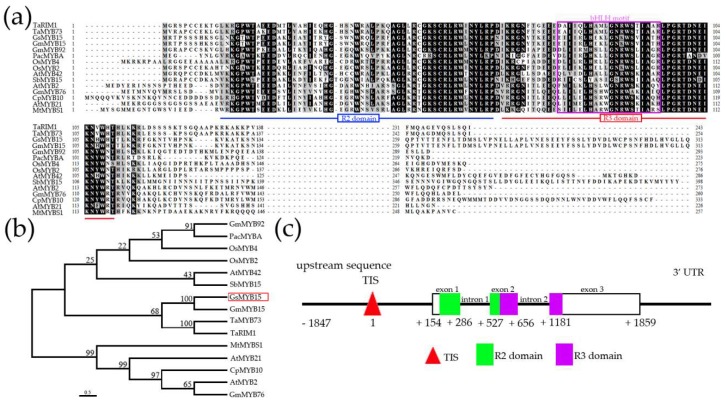
Phylogenetic relationship between the protein sequences of *GsMYB15* and stress-related R2R3 MYBs from other species and genomic sequence analysis of *GsMYB15*. (**a**) Protein sequence alignment of *GsMYB15* with known stress-related R2R3 MYBs from other species. The R2R3-binding domain is underlined. Green boxes indicate specific residues from the motif implicated in the bHLH cofactor interaction in *Arabidopsis* [38]. The accession numbers of these proteins in the GenBank database are as follows: PacMYBA (KF974774), AtMYB42 (AEE83118), AtMYB2 (BAA03534), OsMYB4 (BAA23340), OsMYB2 (BAA23338), CpMYB10 (AAM43912), MtMYBS1 (AES60982), SbMYB15 (AKP07635), TaMYB73 (AEW23186), GmMYB15 (Glyma.12g199200), TaRIM1 (AMP18876), AtMYB21 (EFH51661), GmMYB76 (Glyma.02g009800), GmMYB92 (Glyma.16g023000) and *GsMYB15* (MH796674). (**b**) Phylogenetic tree of *GsMYB15* and other stress-related R2R3 MYB proteins from diverse species. The scale bar indicates 0.5 substitutions per site. (**c**) Structure of the *GsMYB15* gene sequence. The promoter, introns and 3’UTR are indicated by lines. The transcription initiation site (TIS) and the R2R3 MYB domains are shown as triangles and solid boxes, respectively. Numbers refer to the position relative to the TIS of *GsMYB15*.

To clone and characterize the wild soybean MYB TF that plays a crucial role in the response to biotic and abiotic stress, we analyzed the transcriptomic data using ED059 to study cotton bollworm resistance. After analyzing the transcriptomic data, we found that the MYB-like gene was significantly upregulated after cotton bollworm feeding in the resistant soybean cultivar ED059 compared to the control susceptible soybean cultivar. Therefore, we designed a pair of specific primers based on the genomic DNA sequence of ED059 to amplify the MYB gene using PCR. We obtained a single putative R2R3-MYB gene product and the product, which we named *GsMYB15*, shared high homology with the gene AtMYB15. The 939-bp amplification product was the full-length cDNA of *GsMYB15* and was located on chromosome 12 of ED059. The predicted protein *GsMYB15* contained 313 amino acids, with an isoelectric point of 5.62, an aliphatic index of 72.27, an instability index of 41.95 and a calculated molecular mass of 35 kDa (Supplementary Table S2). After alignment of the amino acid sequence of *GsMYB15* with those of other stress-related MYB TFs, *GsMYB15* was shown to contain a typical R2R3-MYB functional domain (Figure 1a) and shared the highest similarity with glycine max *GmMYB15* (100% amino acid identity in the R2R3 domain and 99% identity over the whole coding sequence). Furthermore, a functional motif that is required for the interaction between MYB and bHLH proteins was also identified in the R3 domain of *GsMYB15* (Figure 1a). The phylogenetic tree also demonstrated that *GsMYB15* was closely related to *GmMYB15* (Figure 1b). These results suggested that *GsMYB15* might also have similar functions as R2R3-MYBs, which are known to be related to biotic or abiotic stress.

The *GsMYB15* gene was then amplified from the genomic DNA of ED059 by PCR using specific primers. The gene structure model in Figure 1c revealed that the *GsMYB15* clone, with the approximately 3.7 kb full-length gene sequence, included three exons and two introns. The R2 domain was present in both the first and second exons, while the R3 domain was present in only the second exon (Figure 1c).

Motif analysis of the 1.8-kb region of the promoter sequence of *GsMYB15* revealed seven types of stress-related elements (Table 1). Interestingly, the presence of two MeJA-responsive elements, with the core sequences CGTCA and TGACG, suggested that *GsMYB15* might be involved in the development of insect-plant interactions. Moreover, ARE and O2-site motifs, which are involved in anaerobic induction and the regulation of zein metabolism, respectively, were also identified in the promoter region of *GsMYB15* (Table 1).

### 2.2. Sub-Cellular Localization, Transcriptional Activation Activity and Tissue-Specific Expression of GsMYB15

**Figure 2:**
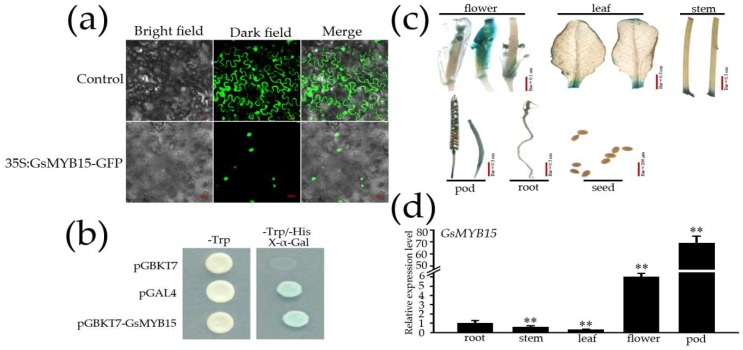
Sub-cellular localization, transcriptional activation, GUS staining and tissue-specific expression of *GsMYB15*. (**a**) Control, transient expression of the PJL12m-GFP vector in tobacco leaves; 35:*GsMYB15*-GFP, transient expression of the PJL12m-*GsMYB15*-GFP vector only in tobacco leaves. (**b**) pGBKT7, negative control; pGBKT7-*GsMYB15*, self-activation of transcription; pGAL4, positive control. (**c**) Histochemical staining of GUS in *Arabidopsis* plants transformed with *GsMYB15*pro::GUS. (**d**) RT-PCR analysis of *GsMYB15* expression in the roots, stems, leaves, flowers and pods. *SKIP16* was used as the internal reference control. Each point represents the mean value of three independent experiments performed in triplicate ± SE. Statistically significant differences were assessed using Student’s *t*-test (** *p* < 0.01).

To assess the sub-cellular localization of *GsMYB15*, we generated a recombinant plasmid containing a fusion of *GsMYB15* with a reporter gene (*GsMYB15*-GFP). This plasmid was introduced into tobacco cells and the fluorescence of the fusion protein, which was visualized by a confocal microscope, was observed to be localized to the nucleus (Figure 2a). This result indicated that *GsMYB15* may function as a TF.

In this study, we used the Y2H Gold yeast system, which harbored four reporter genes (HIS3, ADE2, AUR1-C and MEL1), to detect the transcriptional activation activity of *GsMYB15*. All transformants, including the negative control (pGBKT7), positive control (pGAL4) and experimental group (pGBKT7-*GsMYB15*), grew well on SD/Trp medium (Figure 2B). However, only the positive control and experimental group survived on SD/-Trp/His/X-α-Gal medium and the yeast cells turned blue (Figure 2b). In contrast, the negative control did not grow on SD/-Trp/-His/X-α-Gal medium (Figure 2b). Therefore, our results indicated that *GsMYB15* might have transcriptional activation activity.

To study the tissue localization of *GsMYB15*, *GsMYB15_Pro2000_*-GUS-transformed *Arabidopsis* plants were grown under normal conditions and used for GUS staining. As shown in Figure 2c, *GsMYB15_Pro2000_*-GUS expression occurred in the roots, leaves and stems of transgenic *Arabidopsis* plants and was significantly strong in the pods and flowers. No expression was detected in the seeds.

Expression patterns of *GsMYB15* in different tissues and organs of wild soybean ED059 were detected under normal conditions (Figure 2d). The results showed that *GsMYB15* was expressed in all the organs and tissues examined, including roots, stems, leaves, flowers and pods. *GsMYB15* expression was highest in the pods, followed by flowers, roots, stems and leaves, indicating that *GsMYB15* is expressed at higher levels in reproductive tissues than in nutritive organs and may function as an inducible transcription factor (Figure 2d).

### 2.3. Expression of GsMYB15 in Soybean Leaves in Response to Salt, MeJA and SA Treatments and Insect Attacks

**Figure 3:**
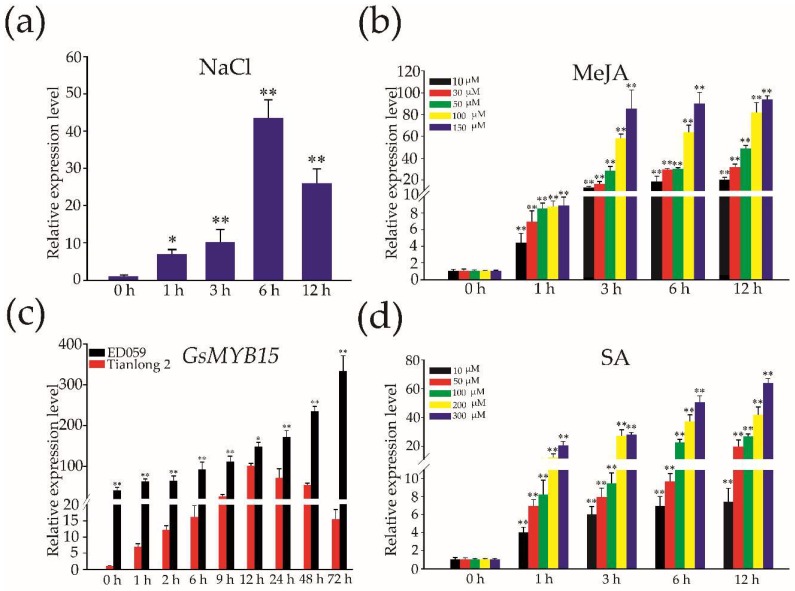
Expression patterns of *GsMYB15* in soybean in response to NaCl (**a**), MeJA (**b**), SA treatments (**c**) and *H. armigera larvae* (**d**). Plants were collected at the indicated time points. *GsSKIP16* was used as the internal reference control to normalize the templates. The relative mRNA levels are represented as the mean ± SD (*n* = 3). Statistically significant differences were assessed using Student’s *t*-test (* *p* < 0.05, ** *p* < 0.01).

To investigate whether *GsMYB15* is involved in salt stress and insect-plant interactions, we examined the expression patterns of *GsMYB15* in the wild soybean ED059 seedlings after exposure to salt stress and to the biotic stress-related plant hormones SA and MeJA. Moreover, we also analyzed the expression patterns of *GsMYB15* in the resistant wild soybean ED059 and susceptible soybean Tianlong 2. The results revealed that salt stress significantly induced *GsMYB15* expression in soybean leaves after 1 h of salt treatment and gradually upregulated expression for up to 6 h of salt treatment, attaining levels that were 4-fold greater than those observed before treatment; the expression levels decreased after 12 h of salt treatment (Figure 3a). QPCR results also showed that *GsMYB15* expression could be induced gradually by both MeJA and SA (Figure 3b). The expression level of *GsMYB15* was also significantly upregulated after *H. armigera* larval attack in the resistant wild soybean ED059 compared with the susceptible soybean Tianlong 2 (Figure 3c). Moreover, the pathogen-related phytohormone SA could induce the expression of *GsMYB15*, similar to MeJA (Figure 3d). These results indicated that *GsMYB15* could respond to salt, MeJA and SA treatments and insect attacks.

### 2.4. Physiological Changes in Arabidopsis Lines Overexpressing GsMYB15

**Figure 4:**
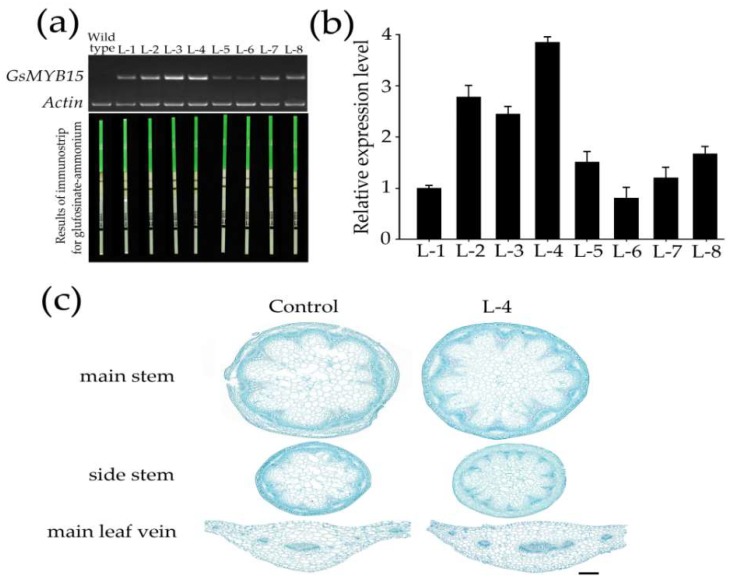
Analyses of *GsMYB15* transgenic plants grown in soil. (**a**) Prove *GsMYB15* transgenic *Arabidopsis* generations using semi-quantitative RT-PCR and immunostrip methods. (**b**) Expression levels of *GsMYB15* in different transgenic *Arabidopsis* lines. *GsSKIP16* was used as the internal reference control. Each point represents the mean value of three independent experiments performed in triplicate ± SE. (**c**) Four-week-old rosette leaves, main stems and side stems from the top were chosen for transverse section analysis. Bar = 200 μm.

To prove the generation of *GsMYB15* transgenic *Arabidopsis* plants, we use PCR method to amplification the coding sequence of *GsMYB15* in both transgenic lines and wild type *Arabidopsis*. As results shown in Figure 4a, bands only exist in agars which using the transgenic generations DNA as amplification templates and no band exist in wild type *Arabidopsis* (Figure 4a). Moreover, the results of immunostrip, which is specifically for detections of the protein of glufosinate-ammonium also demonstrating that only transgenic *Arabidopsis* generations could show two bands while the wild type *Arabidopsis* only have one band (Figure 4a). In this work, three T_3_ *GsMYB15*-overexpressing *Arabidopsis* lines (L-2, L-3 and L-4) with relatively high expression levels were used to investigate the function of *GsMYB15* in plant biotic and abiotic stress responses (Figure 4b). Under normal conditions, no obvious phenotypic differences were observed between the control and transgenic plants. However, the results of paraffin sectioning showed that the transgenic plants (L-4) had more mesophyll cells in the leaf tissues than the control plants (Figure 4c). The results also showed that the transgenic plants had more xylem cell in both the main stem and side stem tissues than the control plants (Figure 4b). The other two transgenic plants (L-2 and L-3) also have the similar phenotypes as L-4 in leaf tissues, main stem and side tissues (date not shown).

### 2.5. Overexpression of GsMYB15 Increased Salt Stress Tolerance in the Transgenic Arabidopsis Lines

**Figure 5:**
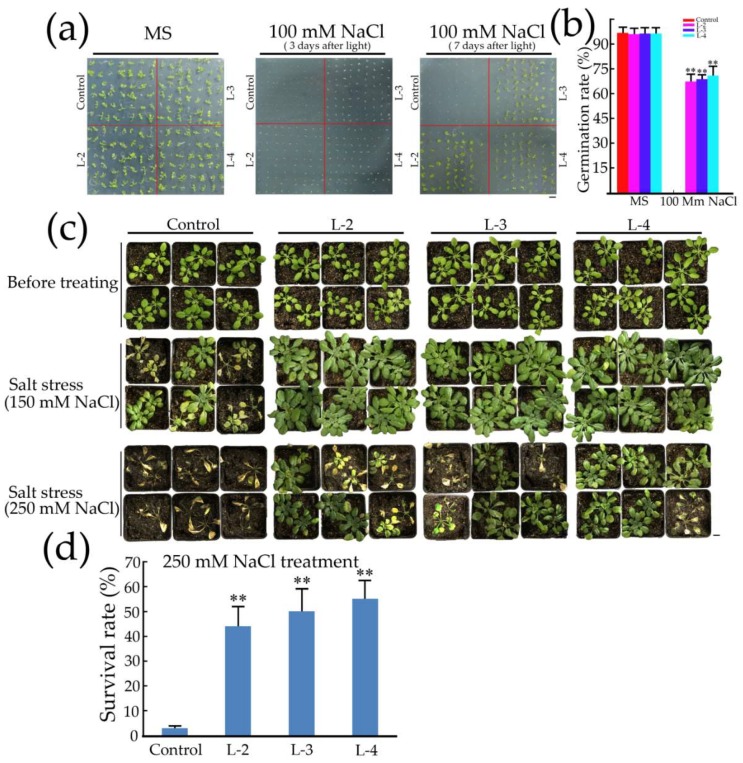
Comparative analysis of seed germination and seedling survival rate under salt stress among control (Col-0) and transgenic *Arabidopsis* plants. (**a**) Germination status of control, L-2, L-3 and L-4 seeds in MS plates, with or without supplementation with 100 mM NaCl, 3 or 7 days after exposure to light. Bar = 1 cm. (**b**) Germination rate of Col-0, L-2, L-3 and L-4 seeds in MS plates, with or without supplementation with 100 mM NaCl, 3 days after exposure to light. (**c**) The phenotypes of the control and transgenic *Arabidopsis* plants under normal and different concentrations (150 mM and 250 mM) of NaCl. Four-week-old seedlings grown in soil were assessed. Bar = 1 cm. (**d**) Survival rates of transgenic *Arabidopsis* plants treated with 250 mM NaCl. Error bars indicate SDs. Statistically significant differences were assessed using Student’s *t*-test (** *p* < 0.01).

To assess the effect of *GsMYB15* overexpression on salt tolerance in *Arabidopsis* plants during the seed germination stage, seeds of control and transgenic plants were sown and germinated on MS medium with or without high-salt treatment. As shown in Figure 5a, there was no phenotypic difference between the control and transgenic seeds during seed germination and seedling development under normal MS medium conditions. After 3 days of treatment in high-salt MS medium (containing 100 mM NaCl), the germination of the control seeds was markedly suppressed (Figure 5a). However, compared to the control seeds, the seeds from the transgenic plants that heterologously expressed *GsMYB15* exhibited markedly increased germination percentages (Figure 5a). Moreover, after 7 days of growth on high-salt MS medium, most of the transgenic plants continued to develop and the leaves remained green after seed germination (Figure 5a). The germination rates of the three transgenic lines treated with 100 mM NaCl ranged from 64 to 71% (Figure 5b).

We next transferred 12-day-old seedlings of the control and transgenic plants from MS medium to soil conditions and grew the seedlings for four weeks to further investigate the salinity tolerance of the transgenic plants during seedling development. Under normal conditions, there were no obvious phenotypic differences between the control and transgenic plants (Figure 5c). After 7 days of treatment with 150 mM NaCl, the control plants exhibited chlorosis in the leaves and growth retardation compared to the transgenic plants (Figure 5c). However, the transgenic plants remained green and grew well after 7 days of treatment (Figure 5c). When treated with 250 mM NaCl, the control plants completely stopped growing and the leaves also turned white and wilted (Figure 5c). In contrast, although the transgenic plants became weak and exhibited leaves with slight chlorosis, most remained alive and continued to grow (Figure 5c). The survival rates of the three transgenic lines treated with 250 mM NaCl ranged from 42 to 53% (Figure 5d). These results indicated that *GsMYB15*-overexpressing *Arabidopsis* lines exhibited enhanced tolerance to salt stress.

### 2.6. Expression Pattern Analysis of Salt-Related Genes in Transgenic Arabidopsis Plants

**Figure 6:**
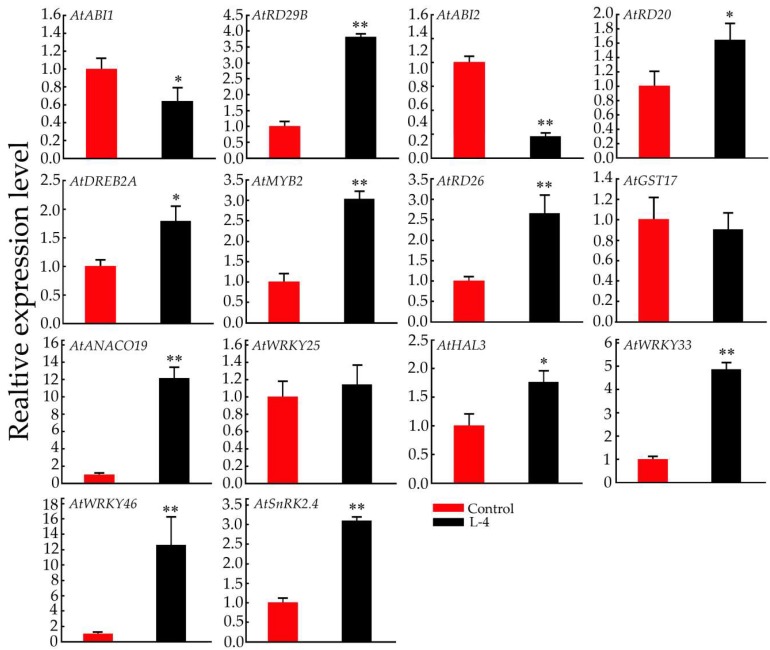
QPCR analysis of the expression levels of salt stress-related genes in the control (Col-0) and transgenic (L-4) *Arabidopsis* plants. *Arabidopsis* actin 1 (*AtActin1*) was used as an internal standard to normalize the templates. The relative mRNA levels are represented as the mean ± SD (*n* = 3). Statistically significant differences were assessed using Student’s *t*-test (* *p* < 0.05, ** *p* < 0.01).

To study the molecular regulatory mechanism of *GsMYB15* in the enhancement of salt tolerance in transgenic *Arabidopsis* plants, we selected several salt-stress-related genes, such as ABA pathway markers (*AtABI1*, *AtABI2* and *AtSnRK2.4*), WRKY TFs (*AtWRKY25*, *AtWRKY33* and *AtWRKY46*) and other salt stress response genes (*AtRD20*, *AtRD26*, *AtRD29B*, *AtDREB2A*, *AtMYB2*, *AtGSTU17*, *AtANACO19* and *AtHAL3*) [1,3,39,40] and detected the expression levels of these genes by real-time PCR (QPCR) and compared the control and transgenic *Arabidopsis* line 4 (L-4). As shown in Figure 6, the expression levels of *AtSnRK2.4*, *AtWRKY25*, *AtWRKY33*, *AtWRKY46*, *AtRD20*, *AtRD26*, *AtRD29B*, *AtDREB2A*, *AtMYB2*, *AtANACO19* and *AtHAL3* were significantly higher in L-4 than in the control plants. The expression levels of AtABI1 and AtABI2 were lower in the transgenic plants than in the control plant. Moreover, the expression of *AtGSTU17* was also lower in L-4 than in the control plants, which indicated that overexpression of *GsMYB15* might weaken glutathione (GSH) biosynthesis and affect several stress-related hormone pathways in transgenic plants. These results indicated that overexpression of *GsMYB15* increased the salt tolerance of transgenic plants by affecting several stress-related hormone signal transduction pathways and the expression of some key stress-related TFs.

### 2.7. Overexpression of GsMYB15 Increased Insect Resistance in Transgenic Arabidopsis Plants

**Figure 7:**
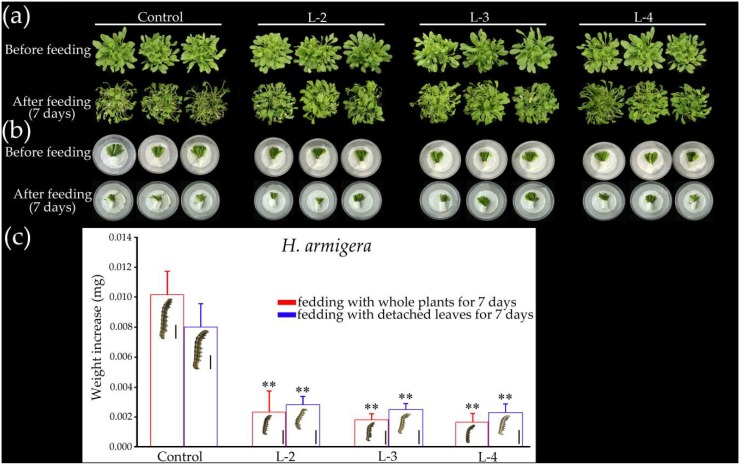
Overexpression of *GsMYB15* enhances insect resistance in transgenic *Arabidopsis* plants. (**a**) Phenotypes of the control plants (Col-0) and *GsMYB15* transgenic plant lines (L-2, L-3 and L-4) before and after *H. armigera* larval attack. (**b**) Phenotypes of detached leaves from the control plants (Col-0) and *GsMYB15* transgenic plant lines before and after feeding with *H. armigera larvae*. (**c**) Weight increase of *H. armigera larvae* fed with whole plants or detached leaves from control plants (Col-0) and *GsMYB15* transgenic plant lines for 7 days, respectively. Data are the means ± SDs (*n* = 15). Shown in the column is an image of an *H. armigera* larva after feeding. Bar = 0.25 cm. Statistically significant differences were assessed using Student’s *t*-test (** *p* < 0.01).

Many studies have shown that JA plays a crucial role in plant resistance to insect invasion [15,41,42]. Based on the results of *cis*-element analysis of the *GsMYB15* promoter, we found six MeJA response elements (Table 1) in the promoter region, indicating that *GsMYB15* may be involved in JA-mediated insect resistance. Moreover, it has been shown (Figure 3b,c) that *GsMYB15* could respond to insect attack and MeJA treatment. Therefore, we tested the insect resistance ability of the control and transgenic plants heterologously expressing *GsMYB15* via a *H. armigera larval* feeding assay. As shown in Figure 7a,b, both the whole plants and detached leaves of the control plants were significantly chewed by *H. armigera*. In contrast, although the transgenic plants were also chewed by the *H. armigera larvae*, the leaf loss was significantly less than that observed for the control plants. The insect feeding assays further showed that *H. armigera larvae* fed transgenic plant leaves gained significantly less weight increase than those fed control plants (Figure 7c). These results indicated that overexpression of *GsMYB15* increased transgenic *Arabidopsis* plant resistance to insects.

### 2.8. Expression Pattern Analysis of Immunity-Related Genes in H. armigera

**Figure 8:**
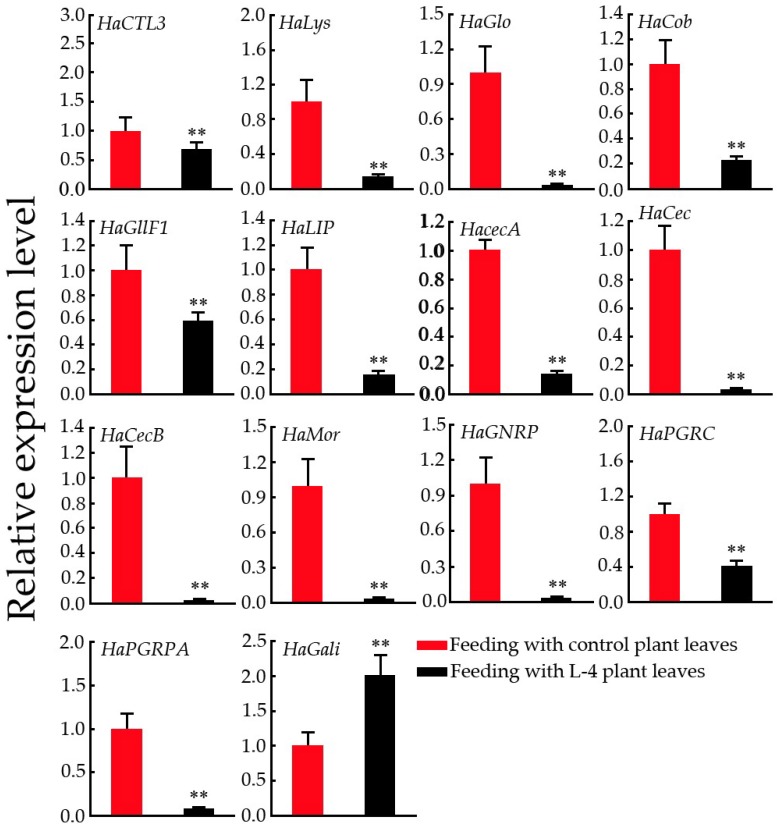
Expression patterns of *H. armigera* immunity-related genes in *H. armigera* fed wild-type plants leaves (red column) and transgenic *Arabidopsis* plant leaves heterologously expressing *GsMYB15* (black column). *H. armigera* actin (HaActin) was used as an internal standard to normalize the templates. The relative mRNA levels are represented as the mean ± SD (*n* = 3). Statistically significant differences were assessed using Student’s *t*-test (** *p* < 0.01).

The above results suggested that overexpression of *GsMYB15* in *Arabidopsis* plants could increase plant resistance to *H. armigera larvae* (Figure 7a,b). The growth of *H. armigera larvae* fed transgenic plant leaves was significantly weaker than that of larvae fed control plant leaves, indicating that the use of transgenic plant leaves as food might affect the digestive and immune systems and induce toxic side effects in *H. armigera larvae*. Therefore, we used qPCR to examine the expression levels of major immunity-related genes in *H. armigera larvae* fed control or transgenic plant leaves. As shown in Figure 8, the expression levels of all the selected immunity-related genes except *HaGali* were significantly higher in the larvae fed control plant leaves than in those fed transgenic plant leaves (Figure 8). These results suggested that the use of transgenic plant leaves as food may severely damage the immune systems of *H. armigera larvae* and further affect normal growth and development.

### 2.9. Expression Pattern Analysis of Defense-Related Genes in Transgenic Arabidopsis Plants

**Figure 9:**
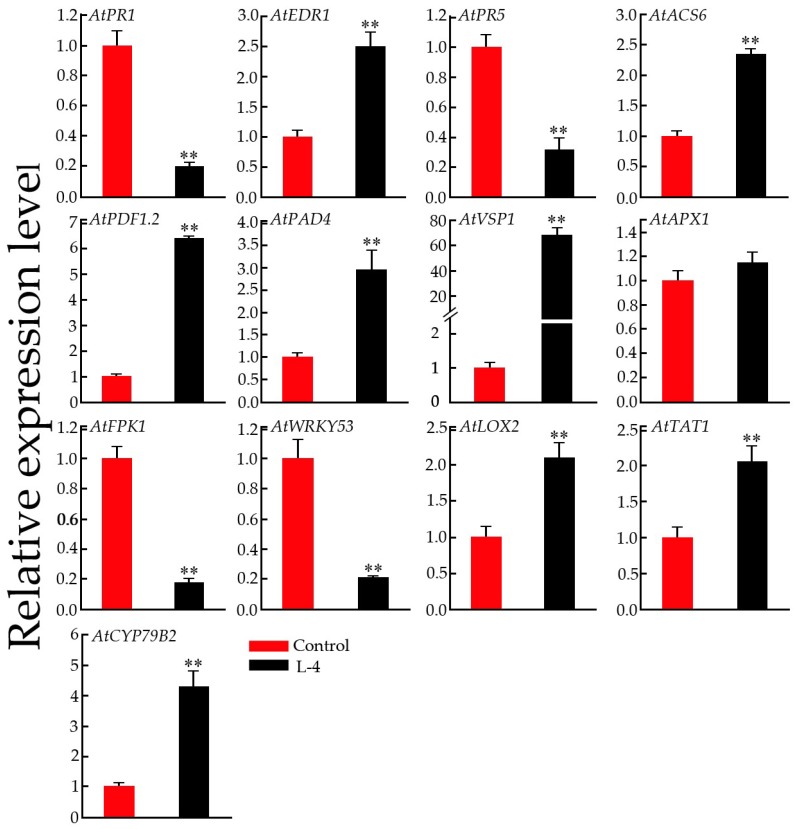
QPCR analysis of the expression levels of defense-related genes in the control (Col-0) and transgenic (L-4) *Arabidopsis* plants. *Arabidopsis* actin 1 (*AtActin1*) was used as an internal standard to normalize the templates. The relative mRNA levels are represented as the mean ± SD (*n* = 3). Statistically significant differences were assessed using Student’s *t*-test (** *p* < 0.01).

To investigate the enhanced resistance of transgenic plants to insect attacks, we examined the expression patterns of PR genes, JA response genes and other defense-related genes. As shown in Figure 9, the results suggested that overexpression of *GsMYB15* could significantly upregulate the expression levels of JA signaling pathway genes, including *AtPDF1.2*, *AtVSP2* and *AtLOX2*. Other defense-related genes, such as *AtEDR1*, *AtACS6*, *AtPAD4*, *AtVSP1*, *AtAPX1*, *AtTAT1* and *AtCYP79B2*, were also upregulated significantly in the transgenic *Arabidopsis* plants compared with the control plants (Figure 9). In contrast, the expression levels of pathogen-related genes (PR1 and PR5) were downregulated in the transgenic *Arabidopsis* plants compared to the control plants (Figure 9). Two negative regulators of stress-related genes were significantly suppressed in transgenic plants compared with control plants.

## 3. Discussion

### 3.1. GsMYB15 Can Respond to Salt Stress, MeJA and SA Treatment and Insect Attacks

Plant MYB-type proteins constitute a large TF family. To date, a number of R2R3-MYB family members have been reported to be involved in the responses of plants to various environmental stress factors in a number of plant species [1,4,10,30]. However, to date, very few soybean R2R3-MYB TFs have been reported to be involved in salt stress and the response to herbivorous insect attacks. In the present study, we isolated and functionally characterized an R2R3-type MYB TF, namely, *GsMYB15*, from the insect-resistant wild soybean ED059. *GsMYB15* was significantly upregulated by NaCl, MeJA and SA treatment. However, previous study demonstrated that exogenous application of SA reduces JA biosynthesis and suppresses JA-mediated gene expression reported that a stress-related R2R3-MYB TF PacMYBA also could be induced by both JA and SA treatments at suitable concentrations [16,43]. Our results suggested that *GsMYB15* might have a similar function to PacMYBA that is involved in stress response. In addition, we identified multiple stress-related cis-elements in the promoter region of *GsMYB15*, suggesting that *GsMYB15* might be regulated by stress signals and other TFs. Furthermore, *GsMYB15* was induced by *H. armigera* larval attack and the expression levels of *GsMYB15* were significantly higher in ED059 than in the susceptible soybean cv. Tianlong 2. These results indicated that *GsMYB15* may be involved in both responses to both abiotic stress and plant-insect interactions.

### 3.2. GsMYB15 Plays a Positive Regulatory Role in Salt Stress Tolerance in Transgenic Arabidopsis Plants

The phytohormone ABA and the ABA signal transduction pathways have been demonstrated to play crucial roles in the plant response to salt stress [6,29]. Plants in high-salt conditions generate primary stress signals followed by secondary signals, such as hyperosmotic signals, leading to the accumulation of ABA, which triggers the ABA signaling pathway and activates the expression of downstream stress response genes, finally leading to enhanced plant salt tolerance [1,12,27]. In our research, heterologous expression of *GsMYB15* in *Arabidopsis* plants significantly improved plant salt tolerance during high-salt irrigation treatment. This result indicated that *GsMYB15* may be associated with the salt stress response. Furthermore, the marker genes (*AtABI1* and *AtABI2*) of negative regulation of the ABA signaling pathway had lower transcript abundance in *GsMYB15* transgenic plants than in the control plants, indicating that *GsMYB15* may be involved in the ABA-dependent pathway under salt stress conditions. In addition, several stress-related TF family genes were also significantly upregulated in the transgenic plants, such as *AtWRKY25*, *AtWRKY33*, *AtWRKY46*, *AtRD20*, *AtRD26*, *AtRD29B*, *AtDREB2A* and *AtMYB2*, all of which have been demonstrated to confer salt stress tolerance in transgenic plants [44,45,46,47,48]. Our results also demonstrated that *GsMYB15* had transcriptional activation activity in Y2H yeast cells, indicating that *GsMYB15* may act as a regulator to regulate the expression of other genes or interacting with other TFs as TF complex to affect downstream genes expression. Taken together, these data indicate that *GsMYB15* may play a positive role in the plant response to salt stress; however, further investigation is required.

### 3.3. GsMYB15 Enhances Herbivorous Insect Resistance in Transgenic Arabidopsis Plants

In field conditions, plants face diverse types of herbivorous insect attacks during the entire plant growth period. Over a long-term evolutionary period, plants have developed sophisticated and integrated regulatory mechanisms to protect themselves from herbivorous insect attacks [15,49]. When the insects chew or suck the plant tissues with their mouth parts, the injured plant cells at the wound produce initial signals, which are then transmitted within the plant via defense-related signal transduction pathways to trigger the plant immune response, finally leading to the promotion of insect resistance [2,14,18]. In these signal transduction networks, TFs play central and essential roles in regulating gene expression by directly or indirectly affecting the promoter regions of target genes [6,10,28]. Lots of studies have suggested that plant R2R3-MYB TFs play crucial roles in both abiotic and biotic stresses. Most studies are founding their important functions on environment stresses and plant disease [50,51,52,53,54]. However, very few studies explore their functions in plant-insect interactions, especially in soybean plants. In the present study, a wild soybean R2R3-MYB TF gene, named *GsMYB15*, we found that the weight increase of *H. armigera larvae* fed *GsMYB15* transgenic plant leaves was less than that of larvae fed control plant leaves, which suggested that heterologous expression of *GsMYB15* could enhance the insect resistance of transgenic plants. Many studies have demonstrated that the JA and SA signaling pathways exhibit antagonistic effects [55]. The JA signaling pathway plays a pivotal role in plant defense against insects [2]. The JA signaling pathway can promote the production and accumulation of defense compounds, including glucosinolates, phenolics, proteinase inhibitors and cyanogenic glucosides, in plants [15,56,57]. The present work showed that heterologous expression of *GsMYB15* could significantly enhance the expression levels of JA response genes such as *AtPDF1.2*, *AtACS6* and *AtPAD4* in transgenic plants compared with control plants. Furthermore, our results also suggested that the expression levels of immunity-related genes were significantly decreased in larvae fed *GsMYB15* transgenic plant leaves for 7 days, indicating that long-term feeding with *GsMYB15* transgenic plant leaves may destroy the immune systems of larvae. Future work should focus on analysis and determination of the primary metabolites that play key roles in insect resistance in *GsMYB15* transgenic plants. Interestingly, paraffin sectioning results showed that there were more mesophyll cells in *GsMYB15* transgenic plant leaves than in the control plants, indicating that these structural changes may have contributed to insect resistance in the *GsMYB15* transgenic plants. 

## 4. Materials and Methods 

### 4.1. Soybean Plants and Treatments

The seeds of the soybean cv. Tianlong 2 and the wild soybean accession ED059 used in this work were obtained from Professor Xinan Zhou (the Institute of Oil Crops Research, Chinese Academy of Agricultural Sciences, Wuhan, China). The seeds were pre-germinated on moistened filter paper in a plant growth chamber at 26 °C with a 16 h light/8 h dark regimen for 5 days. The seedlings were then transferred and grown in Hoagland’s nutrient solution until the third compound leaf opened completely. For salt, SA or methyl jasmonate (MeJA) treatment, the seedlings were placed in a 250 mM NaCl, 10/50/100/200/300 μM SA, or 10/30/50/100/150 μM MeJA solution, respectively and collected after 0, 1, 3, 6, or 12 h. The untreated control contained only Hoagland’s nutrient solution. For *Helicoverpa armigera larval* feeding treatments, the seedlings were collected after 0, 1, 2, 6, 9, 12, 24, 48 or 72 h. The seedlings were immediately frozen in liquid nitrogen and stored at −80 °C until further analysis. All the treatments were performed in biological triplicates.

### 4.2. Sequence Analysis

The 2-kb promoter sequence of *GsMYB15* was amplified by PCR (F, 5′-GGAGATAAAGCAAACTTCTTGTTAC-3′; R, 5′-CTCATATCTGATGCTGTGTTGGC-3′) using the genomic DNA of ED059 as the amplification template. The molecular evolutionary tree and phylogenetic analyses were performed using MEGA, version 5.0 [58]. The stress-related *cis*-acting elements of *GsMYB15* were analyzed using the Plant-CARE database [59]. The protein sequence alignments were first assembled by Clustal W [60] and then, the aligned sequences were edited using BioEdit, version 7.0.4 (http://www.mbio.ncsu.edu/bioedit/bioedit.html). The physicochemical properties of *GsMYB15* were analyzed by the EXPASY online tools (https://web.expasy.org/protparam/).

### 4.3. RNA Isolation and QPCR Analysis

Total RNA was extracted from 0.2 g of fresh soybean leaves (ED059 and Tianlong 2), cotton bollworm, control *Arabidopsis* plants and T3 transgenic *Arabidopsis* plants that heterologously expressed *GsMYB15*. cDNA was synthesized using the First-Strain cDNA Synthesis SuperMix (Transgen Biotech, Beijing, China) according to the manufacturer’s instructions. QPCR was performed as described by [3]. All the primers used for qPCR are listed in Supplementary Table S1. The relative expression level of each gene was quantified using the 2^−△△C*t*^ method [61].

### 4.4. Sub-Cellular Localization and Transcriptional Activation Activity Analysis of GsMYB15

The full-length coding sequence (CDS) of *GsMYB15* was amplified using the following primer pair: F: 5′-ATCTGATCAAGAGACAGGATCCATGAGAACTCCATCATCTTCCTCTC-3′; R: 5′-GCCCTTGCTCACCATGGATCCTTGCAATAAGCCCACGTGCAAATC-3′ (the underlined sequence were the same as the flanking sequences of the insertion cloning site of PJL12m-GFP). The PCR product was sub-cloned into the PJL12m-GFP vector using the LR method (ClonExpress^®^ Entry One Step Cloning Kit, Vazyme, Nanjing, China) to generate PJL12m-*GsMYB15*-GFP. The *GsMYB15*-GFP fusion construct was under the control of the *CaMV35S* promoter. The construct was then transformed into *Agrobacterium* strain EHA105 using the method reported by [62].

*Nicotiana tabacum* ‘benthamiana’ plants were grown in a plant growth chamber at 26 °C with a 16 h light/8 h dark regimen until plants that were approximately 10–20 cm high were available for infiltration with *Agrobacterium* strain EHA105. Infiltration was performed as described by [63]. The agroinfiltrated leaves were photographed 2 days after infiltration. GFP fluorescence images were captured with a Nikon ECLIPSE Ti laser scanning confocal microscope with excitation at 488 nm.

To assess the transcriptional activation activity of *GsMYB15*, the full-length CDS of *GsMYB15* was cloned into the bait vector pGBKT7 (BD) using the following primer pair: F: 5′-AGGAC CTGCATATGGCCATGGAGATGAGAACTCCATCTTCCTCTCACAA-3′; R: 5′-CCGGGAATTCGGCCTCCATGGTCATTGCAATAAGCCCACGTG-3′ (the underlined sequence were the same as the flanking sequences of the insertion cloning site of pGBKT7). The detailed method for the generation of pGBKT7-*GsMYB15* was the same as that for PJL12m-*GsMYB15*-GFP. The vector pGBKT7 (BD) was used as a negative control and the vector pGAL4 was used as a positive control. Yeast two-hybrid (Y2H) assessment was performed as described by [16].

### 4.5. GUS Staining and Expression Analysis

The 2-kb promoter sequence upstream of the start codon (ATG) of *GsMYB15* was amplified from ED059 genomic DNA by PCR using the following primer pair: F: 5′-GACCTGCAGGCATGCAAGCTTCGTTGGGGGTAGATATCGAATC-3′; R: 5′-TTACCCTCAGATCTACCATGGATCTGATGCTGTGTTGGCGATG-3′ (the underlined sequence were the same as the flanking sequences of insertion cloning site of pCambia3301-GUS). The PCR product was then introduced into the pCambia3301 binary vector using the LR method to replace the *CaMV35S* promoter and create GUS fusion constructs. The *_pro_*GsMYB15**::GUS construct was then transformed into wild-type *Arabidopsis* and transgenic plants were selected by resistance to Basta. The T_3_ single-copy insertion transgenic lines were selected by the method described by [62]. The T3 transgenic plants were used for GUS staining as reported by [48].

### 4.6. Generation of GsMYB15 Transgenic Arabidopsis Plants

The full-length CDS of *GsMYB15* was sub-cloned into the binary vector PB2GW7.0 and used to transform *Agrobacterium* strain EHA105. The transgenic *Arabidopsis* plants were transformed with EHA105 by the floral dip method [64]. T_1_ transgenic plants were grown in soil at 21 °C in a plant growth chamber with a 16 h day/8 h night regimen and selected by resistance to Basta. In addition, we also use RT-PCR and immunostrip (Bar Fast Immunostrip Kit, OCRI, Whuhan, China) methods to confirm the generations of *GsMYB15* transgenic *Arabidopsis*. The T_3_ single-copy insertion transgenic lines were selected by the method described by [61].

### 4.7. Tissue Preparation

Paraffin sectioning was performed as described by [65].

### 4.8. Analysis of Salinity Tolerance and Insect Resistance in Transgenic Arabidopsis Plants

For germination analysis, at least 400 seeds per plate from the Control (Col-0), *GsMYB15*-OE2 (L-2), *GsMYB15*-OE3 (L-3) and *GsMYB15*-OE4 (L-4) *Arabidopsis* plants were sown onto MS medium supplemented with 0 or 100 mM NaCl. Germination was assessed from 3 to 7 days after exposure to light. A seed was considered to be germinated when the radical protruded through the envelope. All treatments were performed in biological triplicates.

For salinity stress treatment, five-week-old, nutritional soil-grown, control *Arabidopsis* plants and *Arabidopsis* plants overexpressing *GsMYB15* were irrigated with 0, 150, or 250 mM NaCl solution for 7 days and then with distilled water for 4 days. At the end of the treatment, the leaves were collected and immediately frozen in liquid nitrogen and stored at −80 °C until further analysis. All the treatments were performed in biological triplicates.

For insect resistance analysis, *H. armigera larvae* were hatched at 28 °C from a single egg mass obtained from Huazhong Agricultural University. For whole-plant feeding, each pot of the *Arabidopsis* plants contained fifteen freshly hatched individual *H. armigera larvae* and covered with a mesh bag to contain the larvae. For leaf feeding, detached leaves from control or transgenic plants were placed in a plastic bowl; each plastic bowl contained fifteen freshly hatched individual *H. armigera larvae*. After feeding for 7 days, increases in net weight were recorded. All treatments were performed in biological triplicates.

## 5. Conclusions

In conclusion, we demonstrated that *GsMYB15* is localized to the nucleus and had transcriptional activation activity. Moreover, we describe that the expression of *GsMYB15* could be induced by NaCl, MeJA, SA and *Helicoverpa Armigera* treatments. We also suggested that *GsMYB15* from wild soybean (*Glycine soja*) could increase *Helicoverpa Armigera* resistance and salt tolerance in transgenic *Arabidopsis* plants.

## Figures and Tables

**Table 1:** *Cis*-acting elements potentially associated with the stress response of *GsMYB15*.

Motif	Stand	Distance from ATG	Sequence	Function
ARE	- +	377 1389	TGGTTT TGGTTT	*Cis*-acting regulatory element essential for the anaerobic induction
O2-site	- - -	1049 278 407	GATGATGTTG TTGAGGATGT GTTGACGTGA	*Cis*-acting regulatory element involved in zein metabolism regulation
CGTCA-motif	+ - +	278 407 1119	CGTCA CGTCA CGTCA	*Cis*-acting regulatory element involved in the MeJA-responsiveness
GARE-motif	-	56	TCTGTTT	Gibberellin response element
LTR	+	1135	CCGAAA	*Cis*-acting element involved in low-temperature responsiveness
HSE	+	1460	AAAAAATTTC	*Cis*-acting element involved in heat stress responsiveness
TGACG-motif	- - +	1049 278 407	TGACG TGACG TGACG	*Cis*-acting regulatory element involved in the MeJA-responsiveness

## Supplementary Materials

Supplementary Materials can be found at http://www.mdpi.com/1422-0067/19/12/3958/s1.

## Abbreviations

MeJA	Methyl jasmonate
ABA	Abscisic acid
GUS	Beta-glucuronidase
TF	Transcription factor
SA	Salicylic Acid
bHLH	Basic helix-loop-helix
*H. armigera*	*Helicoverpa armigera*
OE	Overexpression

## References

[1] Zhu J.K. (2002). Salt and drought stress signal transduction in plants. Annu. Rev. Plant Biol..

[2] Howe G., Jander G. (2008). Plant immunity to insect herbivores. Annu. Rev. Plant Biol..

[3] Shen X.J., Guo X.W., Guo X., Zhao D., Zhao W., Chen J.S., Li T.H. (2017). *PacMYBA*, a sweet cherry R2R3-MYB transcription factor, is a positive regulator of salt stress tolerance and pathogen resistance. Plant Physiol. Biochem..

[4] Chen W.J., Zhu T. (2004). Networks of transcription factors with roles in environmental stress response. Trends Plant Sci..

[5] Hirayama T., Shinozaki K. (2010). Research on plant abiotic stress responses in the post-genome era: Past, present and future. Plant J..

[6] Zhu J.K. (2016). Abiotic stress signaling and responses in plants. Cell.

[7] Melcher K., Ng L.M., Zhou X.E., Soon F.F., Xu Y., Suino-Powell K.M., Park S.Y., Weiner J.J., Fujii H., Chinnusamy V. (2009). A gate-latch-lock mechanism for hormone signalling by abscisic acid receptors. Nature.

[8] Walter P., Ron D. (2011). The unfolded protein response: From stress pathway to homeostatic regulation. Science.

[9] Endler A., Kesten C., Schneider R., Zhang Y., Ivakov A., Froehlich A., Funke N., Persson S. (2015). A mechanism for sustained cellulose synthesis during salt stress. Cell.

[10] Nakashima K., Ito Y., Yamaguchi-Shinozaki K. (2009). Transcriptional regulatory networks in response to abiotic stresses in *Arabidopsis* and grasses. Plant Physiol..

[11] Swarbreck S.M., Colaço R., Davies J.M. (2013). Plant calcium-permeable channels. Plant Physiol..

[12] Leng P., Yuan B., Guo Y. (2014). The role of abscisic acid in fruit ripening and responses to abiotic stress. J. Exp. Bot..

[13] Zheng X.Z., Kang S., Jing Y.P., Ren Z.J., Li L.G., Zhou J.M., Berkowitz G., Shi J.S., Fu A.G., Lan W.Z. (2018). Danger-associated peptides close stomata by OST1-independent 3 activation of anion channels in guard cells. Plant Cell.

[14] Yang D.H., Hettenhausen C., Baldwin I.T., Wu J.J. (2012). Silencing nicotiana attenuata calcium-dependent protein kinases, CDPK4 and CDPK5, strongly up-regulates wound- and herbivory-induced jasmonic acid accumulations. Plant Physiol..

[15] Mao Y.B., Liu Y.Q., Chen D.Y., Chen F.Y., Fang X., Hong G.J., Wang W.J., Wang W.J., Chen X.Y. (2015). Jasmonate response decay and defense metabolite accumulation contributes to age-regulated dynamics of plant insect resistance. Nat. Commun..

[16] Shen X.J., Guo X., Zhao D., Zhang Q., Jiang Y.Z., Wang Y.T., Peng X., Wei Y., Zhai Z.F., Zhao W. (2017). Cloning and expression profiling of the PacSnRK2 and PacPP2C gene families during fruit development, ABA treatment, and dehydration stress in sweet cherry. Plant Physiol. Biochem..

[17] Chico J.M., Fernández-Barbero G., Chini A., Fernández-Calvo P., Díez-Díaz M., Solano R. (2014). Repression of Jasmonate-dependent defenses by shade involves differential regulation of protein stability of MYC transcription factors and their JAZ repressors in *Arabidopsis*. Plant Cell.

[18] Louis J., Basu S., Varsani S., Castano-Duque L., Jiang V., Williams W.P., Felton G.W., Luthe D.S. (2015). Ethylene Contributes to maize insect resistance1-Mediated Maize Defense against the Phloem Sap-Sucking Corn Leaf Aphid. Plant Physiol..

[19] Voght S.P., Fluegel M.L., Andrews L.A., Pallanck L.J. (2007). *Drosophila* NPC1b Promotes an Early Step in Sterol Absorption from the Midgut Epithelium. Cell Metab..

[20] Verma V., Ravindran P., Kumar P.P. (2016). Plant hormone-mediated regulation of stress responses. BMC Plant Biol..

[21] Hou X., Lee L.Y., Xia K., Yan Y., Yu H. (2010). DELLAs modulate jasmonate signaling via competitive binding to JAZs. Dev. Cell.

[22] Choi J., Huh S.U., Kojima M., Sakakibara H., Paek K.H., Hwang I. (2010). The cytokinin-activated transcription factor ARR2 promotes plant immunity via TGA3/NPR1-dependent salicylic acid signaling in *Arabidopsis*. Dev. Cell.

[23] Seo P.J., Park C.M. (2010). MYB96-mediated abscisic acid signals induce pathogen resistance response by promoting salicylic acid biosynthesis in *Arabidopsis*. New Phytol..

[24] Li R., Zhang J., Li J.C., Zhou G.X., Wang Q., Bian W.B., Matthias E., Lou Y.G. (2015). Prioritizing plant defence over growth through WRKY regulation facilitates infestation by non-target herbivores. eLife.

[25] Zhou J., Wang J., Zheng Z.Y., Fan B.F., Yu J.Q., Chen Z.X. (2015). Characterization of the promoter and extended C-terminal domain of *Arabidopsis* WRKY33 and functional analysis of tomato WRKY33 homologues in plant stress responses. J. Exp. Bot..

[26] Guo J.P., Xu C.X., Wu D., Zhao Y., Qiu Y.F., Wang X.X., OuYang Y.D., Cai B.D., Liu X., Jing S.L. (2018). Bph6 encodes an exocyst-localized protein and confers broad resistance to planthoppers in rice. Nat. Genet..

[27] Zhong Y.J., Wang Y.G., Guo J.F., Zhu X.L., Shi J., He Q.J., Liu Y., Wu Y.R., Zhang L., Lv Q.D. (2018). Rice SPX6 negatively regulates the phosphate starvation response through suppression of the transcription factor PHR2. New Phytol..

[28] Dubos C., Stracke R., Grotewold E., Weisshaar B., Martin C., Lepiniec L. (2010). MYB transcription factors in *Arabidopsis*. Trends Plant Sci..

[29] Wang T., Tohge T., Ivakov A., Mueller-Roeber B., Fernie A.R., Mutwil M., Schippers J.H., Persson S. (2015). Salt-related MYB1 coordinates abscisic acid biosynthesis and signaling during salt stress in *Arabidopsis*. Plant Physiol..

[30] Wei Q.H., Zhang F., Sun F.S., Luo Q.C., Wang R.B., Chen M.J., Chen M.J., Chang J.L., Yang G.X., He G.Y. (2017). A wheat MYB transcriptional repressor *TaMyb1D* regulates phenylpropanoid metabolism and enhances tolerance to drought and oxidative stresses in transgenic tobacco plants. Plant Sci..

[31] Seo P.J., Lee S.B., Suh M.C., Park M.J., Go Y.S., Park C.M. (2011). The MYB96 Transcription factor regulates cuticular wax biosynthesis under drought conditions in *Arabidopsis*. Plant Cell.

[32] Zhai Y., Li P., Mei Y., Chen M.Y., Chen X.C., Xu H., Zhou X.A., Dong H.S., Zhang C.L., Jiang W.H. (2017). Three MYB genes co-regulate the phloem-based defence against English grain aphid in wheat. J. Exp. Bot..

[33] Onkokesung N., Reichelt M., van Doorn A., Schuurink R.C., van Loon J.J., Dicke M. (2014). Modulation of flavonoid metabolites in **Arabidopsis* thaliana* through overexpression of the MYB75 transcription factor: Role of kaempferol-3,7dirhamnoside in resistance to the specialist insect herbivore *Pieris brassicae*. J. Exp. Bot..

[34] Mourtzinis S., Borg B.S., Naeve S.L., Osthus J., Conley S.P. (2018). Characterizing Soybean Meal Value Variation across the United States: A Swine Case Study. Agron. J..

[35] Olmstead J., Brummer E.C. (2008). Benefits and barriers to perennial forage crops in Iowa corn and soybean rotations. Renew. Agric. Food Syst..

[36] Ullah A., Manghwar H., Shaban M., Khan A.H., Akbar A., Ali U., Ali E., Fahad S. (2018). Phytohormones enhanced drought tolerance in plants: A coping strategy. Environ. Sci. Pollut. Res..

[37] Parmar N., Singh K.H., Sharma D., Singh L., Kumar P., Nanjundan J., Khan Y.J., Chauhan D.K., Thakur A.K. (2017). Genetic engineering strategies for biotic and abiotic stress tolerance and quality enhancement in horticultural crops: A comprehensive review. 3 Biotech.

[38] Espley R.V., Hellens R.P., Putterill J., Stevenson D.E., Kutty-Amma S., Allan A.C. (2007). Red colouration in apple fruit is due to the activity of the MYB transcription factor, MdMYB10. Plant J..

[39] Jakab G., Ton J., Flors V., Zimmerli L., Metraux J.P., Mauch-Mani B. (2005). Enhancing *Arabidopsis* salt and drought stress tolerance by chemical priming for its abscisic acid responses. Plant Physiol..

[40] Chen J.H., Jiang H.W., Hsieh E.J., Chen H.Y., Chien C.T., Hsieh H.L., Lin T.P. (2011). Drought and salt stress tolerance of *Arabidopsis* glutathione S-transferase U17 knockout mutant are attributed to the combined effect of glutathione and abscisic acid. Plant Physiol..

[41] Browse J. (2009). Jasmonate Passes Muster: A receptor and targets for the defense hormone. Annu. Rev. Plant Biol..

[42] Yan C., Fan M., Yang M., Zhao J.J., Zhang W.B., Su Y., Xiao L.L., Deng H.T., Xie D.X. (2018). Injury Activates Ca^2+^/Calmodulin-Dependent Phosphorylation of JAV1-JAZ8-WRKY51 Complex for Jasmonate Biosynthesis. Mol. Cell.

[43] Spoel S.H., Johnson J.S., Dong X. (2007). Regulation of tradeoffs between plant defenses against pathogens with different life styles. Proc. Natl. Acad. Sci. USA.

[44] Abe H.A., Urao T.U., Ito T., Seki M., Shinozaki K., Yamaguchi-Shinozaki K. (2003). **Arabidopsis* AtMYC2* (bHLH) and *AtMYB2* (MYB) Function as transcriptional activators in abscisic acid signaling. Plant Cell.

[45] Jiang Y.Q., Deyholos M.K. (2009). Functional characterization of *Arabidopsis* NaCl-inducible WRKY25 and WRKY33 transcription factors in abiotic stresses. Plant Mol. Biol..

[46] Chung Y., Kwon S.I., Choe S.H. (2014). Antagonistic regulation of *Arabidopsis* growth by brassinosteroids and abiotic stresses. Mol. Cells.

[47] Pruthvi V., Narasimhan R., Nataraja K.N. (2014). Simultaneous expression of abiotic stress responsive transcription factors, *AtDREB2A, AtHB7* and *AtABF3* improves salinity and drought tolerance in peanut (*Arachis hypogaea* L.). PLoS ONE.

[48] Ding Z.J., Yan J.Y., Li C.X., Li G.X., Wu Y.R., Zheng S.J. (2015). Transcription factor WRKY46 modulates the development of *Arabidopsis* lateral roots in osmotic/salt stress conditions via regulation of ABA signaling and auxin homeostasis. Plant J..

[49] Wu J.Q., Baldwin I.T. (2010). New insights into plant responses to the attack from insect herbivores. Annu. Rev. Genet..

[50] An J.P., Li R., Qu F.J., You C.X., Wang X.F., Hao Y.J. (2018). R2R3-MYB transcription factor MdMYB23 is involved in the cold tolerance and proanthocyanidin accumulation in apple. Plant J..

[51] Xu F.C., Liu H.L., Xu Y.Y., Zhao J.R., Guo Y.W., Long L., Gao W., Song C. (2017). Heterogeneous expression of the cotton R2R3-MYB transcription factor GbMYB60 increases salt sensitivity in transgenic *Arabidopsis*. Plant Cell Tissue Organ Cult..

[52] Xie Y.P., Chen P.X., Yan Y., Bao C.N., Li X.W., Wang L.P., Shen X.X., Li H.Y., Liu X.F., Niu C.D. (2017). An atypical R2R3 MYB transcription factor increases cold hardiness by CBF-dependent and CBF-independent pathways in apple. New Phytol..

[53] Guo J.L., Ling H., Ma J.J., Chen Y., Su Y.C., Lin Q.L., Gao S.W., Wang H.B., Que Y.X., Xu L.P. (2017). A sugarcane R2R3-MYB transcription factor gene is alternatively spliced during drought stress. Sci. Rep..

[54] Li T., Zhang X.Y., Huang Y., Xu Z.S., Wang F., Xiong A.S. (2018). An R2R3-MYB transcription factor, S1MYB28, involved in the regulation of TYLCV infection in tomato. Sci. Hortic..

[55] Spoel S.H., Koornneef A., Claessens S.M.C., Korzelius J.P., Van Pelt J.A., Mueller M.J., Bechala A.J., Metraux J.P., Brown R., Kazan K. (2003). NPR1 Modulates Cross-Talk between Salicylate- and Jasmonate-Dependent defense pathways through a novel function in the cytosol. Plant Cell.

[56] Wasternack C. (2007). Jasmonates: An update on biosynthesis, signal transduction and action in plant stress response, growth and development. Ann. Bot..

[57] Koo A.J., Gao X., Jones A.D., Howe G.A. (2009). A rapid wound signal activates the systemic synthesis of bioactive jasmonates in *Arabidopsis*. Plant J..

[58] Tamura K., Peterson D., Peterson N., Stecher G., Nei M., Kumar S. (2011). MEGA5: Molecular evolutionary genetics analysis using maximum likelihood, evolutionary distance, and maximum parsimony methods. Mol. Biol. Evol..

[59] Lescot M., Déhais P., Thijs G., Marchal K., Moreau Y., Peer Y.V. (2002). PlantCARE, a database of plant *cis*-acting regulator elements and a portal to tools for in silico analysis of promoter sequences. Nucleic Acids Res..

[60] Chenna R. (2003). Multiple sequence alignment with the Clustal series of programs. Nucleic Acids Res..

[61] Livak K.J., Schmittgen T.D. (2001). Analysis of relative gene expression data using real-time quantitative PCR and the 2^−∆∆C*t*^ Method. Methods.

[62] Shen X., Zhao K., Liu L., Zhang K., Yuan H., Liao X., Wang Q., Guo X.W., Li F., Li T.H. (2014). A role for *PacMYBA* in ABA-regulated anthocyanin biosynthesis in red-colored sweet cherry cv. Hong Deng (*Prunus avium* L.). Plant Cell Physiol..

[63] Bai G., Yang D.H., Zhao Y., Ha S., Yang F., Ma J., Gao X.S., Wang Z.M., Zhu J.K. (2013). Interactions between soybean ABA receptors and type 2C protein phosphatases. Plant Mol. Biol..

[64] Steven J.C., Andrew F.B. (1998). Floral dip: A simplified method for Agrobacterium-mediated transformation of **Arabidopsis* thaliana*. Plant J..

[65] Kerk N.M., Ceserani T., Tausta S.L., Sussex I.M., Nelson T.M. (2003). Laser capture microdissection of cells from plant tissues. Plant Physiol..

