# Supplementary material for: Overexpression of the Wild Soybean R2R3-MYB Transcription Factor GsMYB15 Enhances Resistance to Salt Stress and Helicoverpa Armigera in Transgenic Arabidopsis
Source: Int J Mol Sci. 2018 Dec 9;19(12):3958. doi: 10.3390/ijms19123958 (PMC6321161; doi:10.3390/ijms19123958)
Supplement: Supplementary file 1 [file ijms-19-03958-s001.pdf]

Supplementary materials

Supplementary Table S1. Primers used for real – time RT-PCR.

Gene name	Forward primer (5′ to 3′)	Reverse primer (5′ to 3′)	Product (bp)
GsMYB15 (MH796674)	ATATCACTCGTTATGGCTCTTGG	TAGTTCCCTCTTTTTATGTTGGG	127
GmMYB15(Glyma.12g199200)	ATATCACTCGTTATGGCTCTTGG	TAGTTCCCTCTTTTTATGTTGGG	127
SKIP16(Glyma.12g05510)	GAGCCCAAGACATTGCGAGAG	CGGAAGCGAAGAACTGAACC	
AtABI1 (AT4G26080)	GTTTTCCCGTCTCACATCTTCGT	CTTCATCCGTCATTACATCCCAA	325
AtABI2 (AT5G57050)	TTGTCTTTCCGACTCACATCTTT	CTTTTCCTTCTCCTCTTTTCTCC	429
AtRD29B (AT5G52300)	GAGGATCGGATTATCTCAGTGGT	TCTGTGTAGGTGCTTGGTTTTGT	439
AtRD20 (AT2G33380)	ATCGACAACATACACAAAGCCAA	TCCATCAAAGCAACCTCTCACAG	285
AtDREB2A (AT5G05410)	TGGAAAGAGTATAACGAGACCGT	CCTCATCATAAGCAGAAGCAGCT	247
AtMYB2 (AT2G47190)	TTCAATTCAGCCAGAATCATCAT	AAAAACTCTCCTCGTCAGTCCAC	213
AtRD26 (AT4G27410)	CATGTTTCACATTTGTAGGGGAG	CGAGGAAGATTAAAAGACTGGTC	499
AtGSTU17 (AT1G10370)	TCTAAACCTCAAGTCTGTCCCCT	GCTTTCTTCTCTTCTTCTCCTCC	309
AtSnRK2.4 (AT1G10940)	TCTCCTTTCCCGTATATTTGTCG	TATGCAGCTTGAGCTGTCTCTGT	123
AtHAL3 (AT1G48605)	TAGCTGCAAGTGGAAGTGTGG	TTGATGGGAGGAATTAGGGTG	439
AtANAC019 (AT1G52890)	AAGAAGCAATCAAGTGCACAAAA	TTCAAAGTGAGAAGAAGACGACG	111
AtWRKY25 (AT2G30250)	CTCCGATTACCTTGATTCTCCTC	TTTTCGCTCTTCTTCACTTGTTT	357
AtWRKY33 (AT2G38470)	TTTTTCTCGATTCCCCTGCTT	CTCGGTTTGGCTCCATTGTTC	293
AtWRKY46 (AT2G46400)	TAACAACATCACATCCCCGAAGA	GCAGCAGGTGCAGATAAAACCTC	315
AtActin2 (NM_180280)	GGATCTGTACGGTAACATTGTGC	AACCACCGATCCAGACACTGT	982
HaPGRPA (GU182905)	CCAGATGTGCTGAGATCGTG	TTTGCCGTTACCACCTACAA	94
HaPGRC (GU182906)	AGCTGAGGTACCCACAGACG	CCAGCCCAAGCTGTTATGAT	143
HaGNRP (GU182914)	CACCTTGCATTACGGACCTT	TGTTACACGTCCCAGTTCCA	159
HaGali (GU182907)	TGGTGAAGAGCTACCGTTCC	ACGCAGCTACCAATCAGCTT	102
HaGlo (GU182908)	GCAAGACATCTTCAACGACCA	TCCTTGTACACATCAAGACTGG	140
HaLys (GU182915)	GAAGGACTGCAATGTTACTTG	GCCTCGAACTTGTGGCGTTTG	90
HaCec (GU182916)	GTTTGGTAGCAGCGTGCAG	GCTTCACCGAGGACTGCTAT	130
HaCecA (GU182909)	TGTCTTCGCTTGTTTTGTGG	ATCACGAATGTGCTGACCAA	93
HaCecB (GU182910)	GTTGTTCGTGTTCGCGTGT	ACCGTCCCTGATGTTACGAC	104
HaMor (GU182911)	GCATTACTGGTGCCATCTGA	CTATGTTGATCGCCCGGAGT	95
HaCob (GU182912)	TGTGCTAGTTGTTATAAGTGCCATT	CTACCTGCACCGAGTTGTCA	117
HaGallF1 (GU182913)	ACAAGGGCCACCTCTTCCAG	AAGTGCAGTATCCGCCAGAC	84
HaLIp (DQ875243)	CTTATAGGGTGCGACCAACG	ACGTCCGAGTTACAGCGAAG	604
HaCTL3 (JQ250828)	GATGTAAGGTGGAGGGAGCA	GCAGGTTCCTCAGCAGAATC	141
Ha18s (AB620126)	GCATCTTTCAAATGTCTGC	TACTCATTCCGATTACGAG	219
AtPR1 (AT2G14610)	TCTTCCCTCGAAAGCTCAAGA	AGACAAGTCACCGCTACCCCA	211
AtPR5 (AT1G75040)	TCAGAACAATGTCGTGGCCTG	CTCGTTTCGTCGTCATAAGCG	171
AtEDR1 (AT1G08720)	GCTTACACACCAGTACACCGACA	CTAGTTCCCAAAGTATTACCCCG	243
AtACS6 (AT4G11280)	TAGATGGTTTAGGGATTGGTTGG	GACACGTTGAGCTTCACTTGGTG	145
AtPDF1.2 (AT5G44420)	CATCACCCTTATCTTCGCTGC	AACCCCTGACCATGTCCCACT	109
AtPAD4 (AT3G52430)	GGAAACCAATCTCTCTCCACCTC	ACATTCTTTGACTAAAACGCCAC	399
AtAPX1 (AT1G07890)	GAAGAGAGGACAAGCCCCAAC	CAGCGTAATCAGCGAAAAAGG	357
AtVSP1 (AT5G24780)	CGCTCTATCTCCATTTCACGA	ATCCTCAACCAAATCAGCCCA	425
AtFRK1 (At2g19190)	GCCAACGGAGACATTAGAG	CCATAACGACCTGACTCATC	219
AtVSP1 (At5G24780)	CGCTCTATCTCCATTTCACGA	ATCCTCAACCAAATCAGCCCA	276
AtTAT1 (At5G53970)	CCCTCAAAGACGTCAATGGT	ACACGACACGACAAGTCCAA	273
AtLOX2 (At3G45140)	TTGGTGTGGTAACTACGATTGC	CACCAGCTCCAGCTCTATTCTT	417
AtWRKY53 (At4G23810)	CACCAGAGTCAAACCAGCCATTAC	CTTTACCATCATCAAGCCCATCGG	101
AtCYP79B2 (At4G39950)	TAACGGTCACGAGAAGATTATG	GAGTTCTCTTTCCTTCTCTCCA	107

Supplementary Table S2. Physicochemical property analysis for GsMYB15.

Gene name	Gene ID	Amino acid residues	MW (Da)	pI	instability index	Aliphatic index	GRAVY	Chromosome
GsMYB15	MH796674	313	35,459.52	5.62	41.95	72.27	-0.607	12
